# Control of non-volatile magnetic properties of Fe/CoO grown on a piezoelectric substrate

**DOI:** 10.1038/s41598-025-31017-x

**Published:** 2025-12-10

**Authors:** W. Janus, M. Szpytma, E. Oleś, A. Kwiatkowski, P. Dróżdż, J. Kanak, M. Zając, M. Ślęzak, T. Ślęzak, A. Kozioł-Rachwał

**Affiliations:** 1 Faculty of Physics and Applied Computer Science, AGH University of Krakow, Krakow, Poland; 2 AGH University of Krakow, Institute of Electronics, Krakow, Poland; 3 National Synchrotron Radiation Centre SOLARIS, Jagiellonian University, Krakow, Poland; 4 Present Address: Institut de Ciència de Materials de Barcelona (ICMAB-CSIC), Campus de la Universitat Autònoma de Barcelona, Bellaterra, 08193 Spain; 5 Present Address: National Synchrotron Radiation Centre SOLARIS, Jagiellonian University, Krakow, Poland

**Keywords:** Materials science, Nanoscience and technology, Physics

## Abstract

In this study, we investigate the piezoelectric modulation of magnetic properties in an Fe/CoO bilayer grown on a Cr-buffered PMN-PT(001) substrate. An initial application of an electric field across the substrate has induced a non-volatile change in the coercive field of Fe/CoO. Subsequent voltage applications further modified the coercive field of Fe/CoO, maintaining the non-volatile effect below the blocking temperature of CoO. In contrast, no piezoelectric influence on the magnetic properties was observed in the Fe single layer grown on Cr/PMN-PT. X-ray magnetic linear dichroism measurements confirmed that the piezoelectric-driven modulation of magnetic behavior in Fe/CoO bilayer is primarily governed by changes in the magnetic state of the CoO film.

**Supplementary Information:**

The online version contains supplementary material available at 10.1038/s41598-025-31017-x.

## Introduction

Controlling magnetic properties through an electric field has recently emerged as a promising strategy for developing low-power spintronic technologies^1^. An effective approach utilizes electric-field-induced strain from a ferroelectric (FE) substrate to modulate the magnetic state of adjacent thin films. The insulating nature of ferroelectric materials helps to minimize Joule heating, making piezoelectric strain control a highly attractive method for low-power writing applications. The inverse piezoelectric effect alters the size and polarization direction of FE domains, leading to changes in the dielectric, mechanical, and optical properties of the substrate. These modifications can, in turn, influence the properties of the layers grown on the substrate. In particular, piezoelectric strain generated within the substrate can be transferred to the magnetic layer via magnetostriction, enabling control of its magnetic properties.

Among ferroelectric substrates, Pb(Mg_1/3_Nb_2/3_)O_3_-PbTiO_3_ (PMN-PT) is particularly valuable due to its exceptional piezoelectric and dielectric coefficients^2–4^, which enable significant strain generation and effective control over adjacent layers. In a rhombohedral PMN-PT crystal, polarization is aligned along the <111> pseudo-cubic directions. Consequently, an electrically-driven change in polarization involves the rotation of the polarization vector by 71°, 109°, or 180°. When an electric field is applied along the [001] direction to a PMN-PT substrate with a (001) orientation, two types of polarization switching can be observed: volatile and non-volatile. Switching by 71° and 180° results in symmetric, butterfly-like in-plane strain-electric field (S-E) behavior without remanent strain^5^. In contrast, a 109° polarization switch induces reversible, non-volatile strain, resulting in a loop-like bipolar strain response^6,7^. This non-volatile strain is especially attractive from an application point of view, since it enables a memory effect in magnetic material/piezoelectric heterostructures. The effect of applying voltage to a PMN-PT crystal depends on its composition, the direction of the electric field relative to the crystal’s orientation and its initial state^4,8^. In the virgin state, the polarization domains of FE crystal are randomly oriented. The first application of voltage along the non-polar axis can cause irreversible reorientation of these domains into a stable multidomain state known as 4R. This process, referred to as the ‘poling’ process, establishes a preferred polarization direction in the crystal.

The realization of electric-field control of magnetism using PMN-PT has been primarily demonstrated in heterostructures with a ferromagnetic (FM) layer deposited on FE substrates. The mechanisms responsible for the magnetoelectric coupling in FM/PMN-PT systems include charge accumulation or depletion at the interface, strain effects, ion migration, and changes in the morphology of the PMN-PT substrate^1,9–11^. Electric-field induced modulation of magnetic properties was also presented for antiferromagnet (AFM)/FM bilayers grown on PZT^12,13^, PZN-PT^14^, and BaTiO_3_ FE substrates^15^. In the aforementioned studies, a volatile piezoelectric manipulation of exchange bias was reported and linked to the modulation of magnetic anisotropy in the FM film. More recently, voltage-induced strain was proposed as an effective method to control antiferromagnetism^16^. Wu et al. showed that strain-mediated electric modulation of exchange bias in the CoFe/BiFeO_3_/SrRuO_3_/PMN-PT heterostructure is primarily driven by alterations in the AFM spin structure^17^. Piezoelectric strain control of NiO was presented in Ni/NiO/PMN-PT, NiO/Ni/PMN-PT and Pt/NiO/Pt/PMN-PT heterostructures^18–20^. Notably, Domann et al. showed that different strain ranges can be used to modulate spin structure of AFM and FM in NiO/Ni/PMN-PT^19^. Recently, piezoelectric strain has been employed to control magnon spin-current transmission through the AFM insulator in Y₃Fe₅O₁₂/Cr₂O₃/Pt. Zhou et al. demonstrated that non-volatile manipulation of magnon transport can be achieved by adjusting the relative orientation between the Néel vector and spin polarization, which is modulated by ferroelastic strain from the PMN-PT^21^. Possibility of piezoelectric control of AFM spins was also examined for metallic AFM. Switching of AFM spin structure was presented in Mn_3_Pt/BaTiO_3_^22^, MnPt/PMN-PT^23^, Mn_2_Au/PMN-PT^24^, and FeRh/PMN-PT(BaTiO_3_)^25–27^.

Promising candidates for piezoelectric switching are materials with large magnetostriction. A representative insulating AFM with considerable magnetostriction is CoO^28^. Recently, strong sensitivity of its magnetic properties to mechanical bending was demonstrated for Co/CoO grown on a mica substrate^29^, where strain-induced modulation of the bilayer’s magnetic state was attributed to changes in the CoO spin structure. This observation is consistent with earlier studies showing epitaxial strain-driven reorientation of AFM spins in CoO^30^. Despite these indications, systematic exploration of piezoelectric strain control in CoO-based heterostructures remains limited. In particular, the role of CoO in mediating non-volatile strain effects and their impact on exchange coupling in FM/AFM bilayers has not yet been clarified. Motivated by this gap, the present work investigates Fe/CoO bilayers grown on Cr-buffered PMN-PT(001), focusing on how electric-field-induced strain influences the coercive field and magnetic state of the system.

## Experimental

**Fig. 1:**
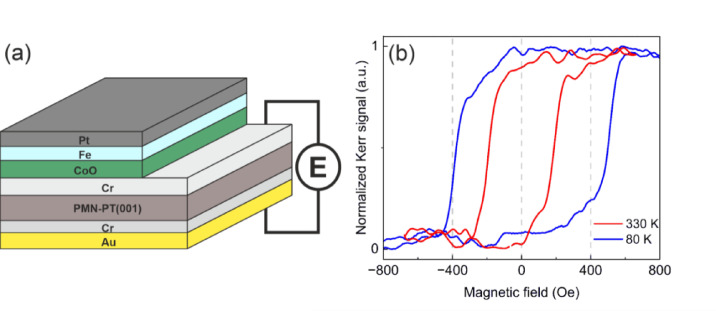
(**a**) Schematic of the sample. (**b**) LMOKE magnetic hysteresis loops acquired at 330 K (red curve) and 80 K (blue curve).

Samples were grown in an ultrahigh vacuum (UHV) chamber using molecular beam epitaxy (MBE) on double-side polished PMN-32%PT(001) substrates with dimensions of 5 mm × 10 mm × 0.5 mm. The substrates used in this study were purchased from Atom Optics Co., Ltd. (Shanghai, China). Prior to deposition, the substrates were annealed at 523 K for 40 min. A 20 nm-thick Cr layer was deposited on one side of the substrate at 473 K, serving as the top electrode. The Cr layer was partially covered with a 10 nm-thick CoO layer, grown at 473 K by reactive deposition of metallic Co under an oxygen partial pressure of 1 × 10⁻⁶ mbar. The area of the sample uncoated with CoO provided access to the Cr electrode. Next, at room temperature (RT), a 5 nm-thick Fe layer was deposited and capped with a 3 nm-thick Pt layer. On the opposite side of the substrate, a bottom electrode consisting of 10 nm of Cr and 60 nm of Au was deposited. A schematic of the sample is shown in (Fig. 1a).

The magnetic properties of the samples were characterized using longitudinal magneto-optic Kerr effect (LMOKE). The setup contained a lock-in detection system consisting of an s-polarized laser (wavelength λ = 635 nm) and a photo-elastic modulator operating at a modulation frequency of 50 kHz. The detector measured the second harmonic signal (2f), which is directly related to the Kerr rotation. The structural properties of the piezoelectric substrate were characterized using an X-ray diffractometer (XRD, X’Pert MPD) equipped with Cu Kα radiation. The X-ray diffraction analysis (Supplementary Material Fig. S1) confirmed the crystalline structure of the substrate. Only peaks corresponding to (001)-oriented PMN-PT were detected, with no evidence of additional peaks, which suggest a polycrystalline nature of the evaporated layers.

X-ray magnetic linear dichroism (XMLD) measurements were conducted at PIRX beamline^31^ of the National Synchrotron Radiation Center SOLARIS^32^. The spectra were recorded in total electron yield (TEY) detection mode. To probe the spin orientation in the CoO layer, linearly polarized X-rays at the photon energy corresponding to the Co L₃ edge were used.

## Results and discussion

We first focus on the MOKE measurements performed on the Fe/CoO/Cr/PMN-PT(001) system in the virgin state, which refers to the condition of the sample before the first application of an electric field to the piezoelectric substrate. Azimuthal angular-dependent MOKE measurements (Supplementary Material Fig. S2) revealed negligible anisotropy in the system, therefore, all MOKE hysteresis loops were recorded with the external magnetic field applied along the in-plane [100] direction of the PMN-PT substrate. Figure 1b shows LMOKE hysteresis loops measured at 330 K (red curve) and 80 K (blue curve) for the sample in the virgin state. The loop at 80 K exhibits more than twice the coercivity of the loop at 330 K and is horizontally shifted towards positive magnetic field values, indicating the presence of the exchange bias effect. The enhanced coercivity and exchange bias at low temperatures are attributed to the formation of antiferromagnetic order in CoO layer upon cooling the system, along with the establishment of interfacial coupling between FM and AFM spins. Notably, the exchange bias in Fig. 1b is observed under zero-field cooling (ZFC), as no difference in the hysteresis loops was detected between cooling under an external magnetic field and cooling in zero field. Furthermore, no training effect was observed in our sample (see Supplementary Material Fig. S3).

**Fig. 2:**
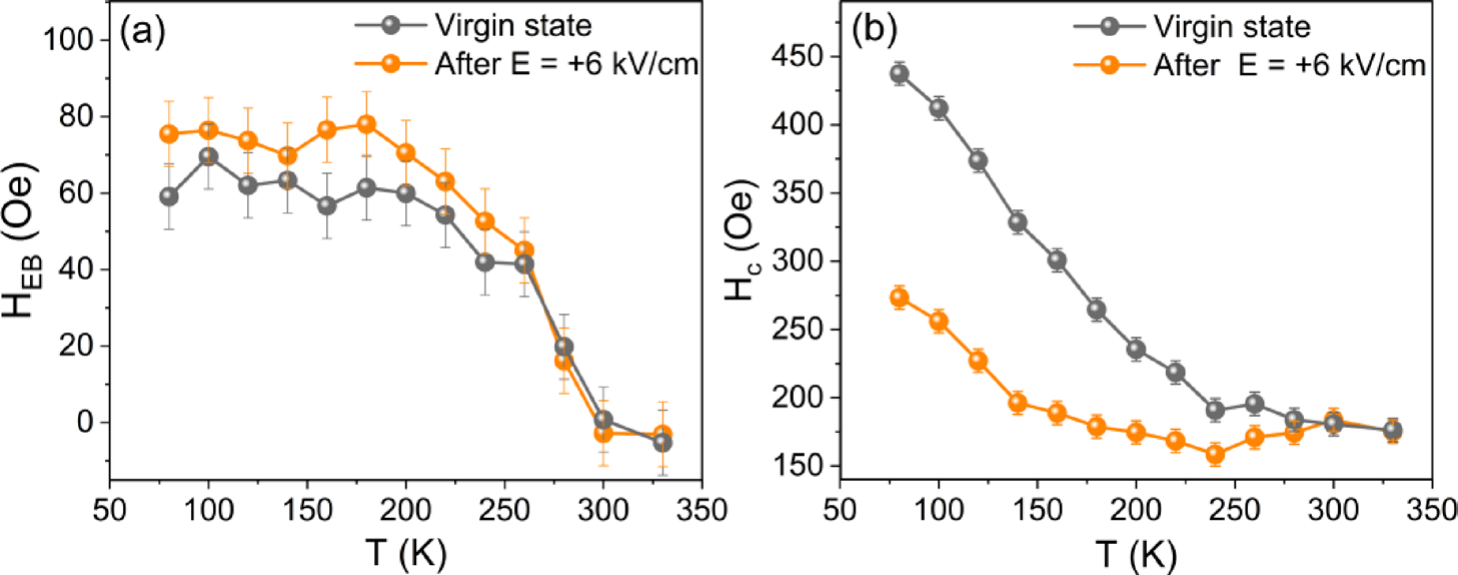
(**a**) Exchange bias field (*H*_EB_) and (**b**) coercive field (*H*_c_) as a function of the temperature (T) for the virgin state of the sample (grey points) and after applying a positive electric field to the substrate (orange points).

Next, we investigate whether the piezoelectric response influences the magnetic properties of the system. Figures 2a,b present temperature evolution of the exchange bias field (*H*_EB_ = (*H*_c1_+*H*_c2_)/2) and coercive field (*H*_c_ = (*H*_c1_-*H*_c2_)/2) for both the virgin state and after applying an electric field, respectively. The post-electric field state corresponds to the sample condition after the first application of an electric field to the substrate. An electric field of + 6 kV/cm (equivalent to a voltage of + 300 V) was applied along the PMN-PT[001] direction at room temperature. As seen in Fig. 2a, *H*_EB_ displays only a minor variation between the virgin and post-electric-field states. For both sample states, the blocking temperature (T_B_), defined as the temperature at which exchange bias disappears, is approximately 300 K. In contrast, a more pronounced effect of the applied electric field is observed in *H*_c_, as shown in Fig. 2b. At lower temperatures, the electric field leads to a significant reduction in *H*_c_, particularly at 80 K, where it decreases by 164 Oe (36%) compared to the virgin state. The difference in *H*_c_ between the two states gradually decreases as the temperature increases, eventually disappearing at 300 K (see Supplementary Material Fig. S4). The temperature at which the difference in coercivities is diminished coincides with the blocking temperature of the system (Fig. 2a) and is slightly higher than the Néel temperature of bulk CoO, T_N_ = 293 K^33^. This suggests that the electric field no longer affects *H*_c_ at the temperature where the AFM order in the CoO layer is either lost, or the magnetic anisotropy within the CoO layer becomes too weak to significantly influence the coercivity of the adjacent Fe layer. Therefore, the observed field-induced changes in magnetic properties of the system are likely related to modulations in the magnetic state of the CoO layer. These modulations can further affect the magnetic properties of the Fe layer through the AFM/FM coupling at the interface, resulting in a reduction of *H*_c_ in the Fe/CoO bilayer.

**Fig. 3:**
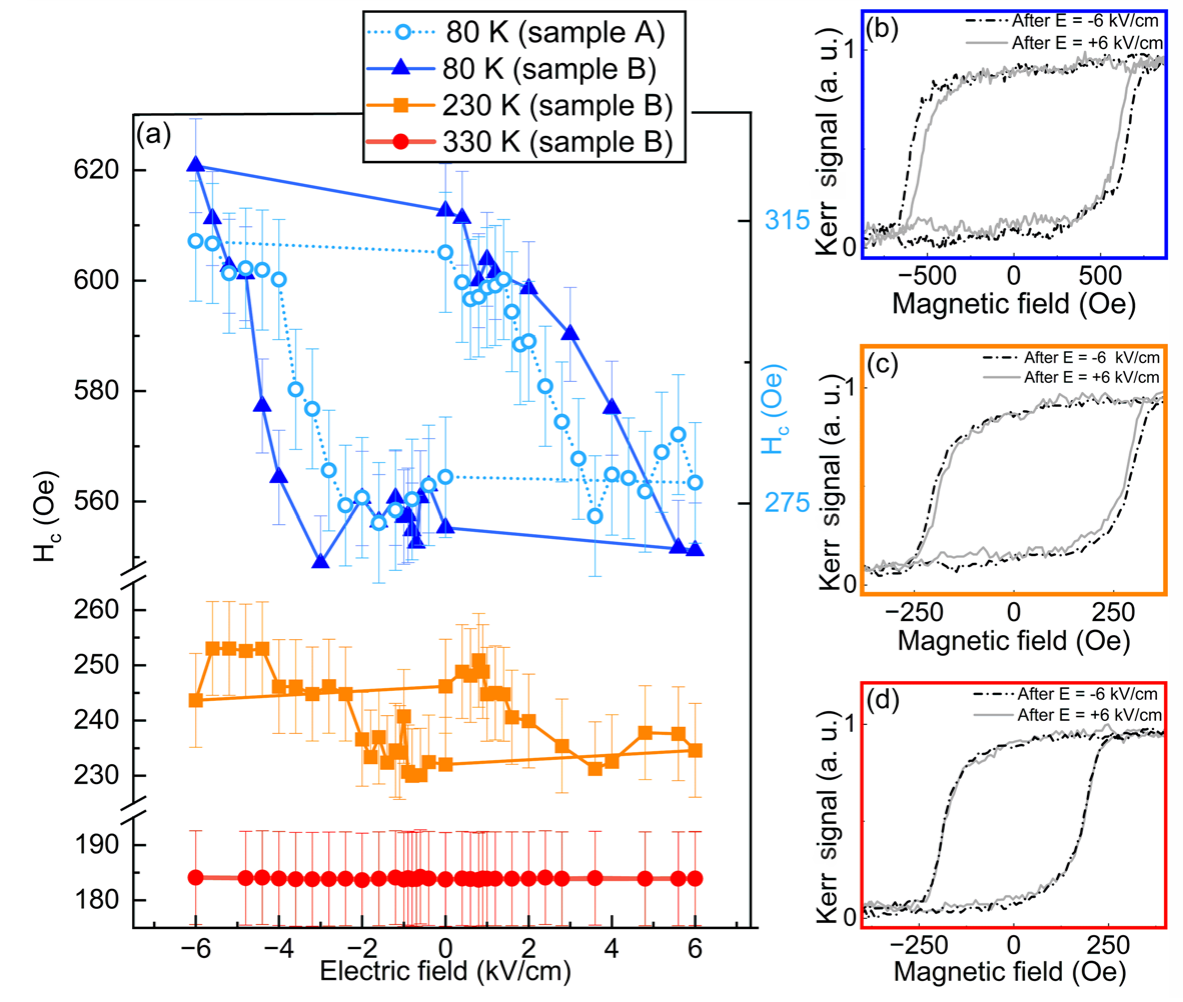
(**a**) Coercive field (*H*_c_) as a function of the applied electric field at 80 K (dark blue triangles, light blue circles), 230 K (orange squares), and 330 K (red circles). Samples A and B have nominally identical architecture (see text). (**b**–**d**) LMOKE hysteresis loops measured at zero electric field after applying + 6 kV/cm (grey curves) and − 6 kV/cm (black dashed curves) measured at 80 K, 230 K, and 330 K, respectively.

Figure 2b shows that the electric-field-induced change in coercivity is largest at 80 K; therefore, we performed MOKE measurements during a bipolar electric field cycle at this temperature. Figure 3a, light blue circles, shows the strong dependence of *H*_c_ on the applied electric field. When an electric field of E = − 6 kV/cm is applied, *H*_c_ increases by approximately 12% compared to the value at E = + 6 kV/cm. A comparable 12% increase in *H*_c_ under E = − 6 kV/cm is observed for a new twin sample “B” (Fig. 3a, dark blue triangles), which was examined following the mechanical failure of the initial sample “A”. For both samples we did not observe any modulation of exchange bias as a function of electric field (not shown). For sample “B” part of the substrate was protected by a shutter during the CoO layer growth. As a result, regions with Fe/Cr and Fe/CoO were formed on the sample. Although the relative change in *H*_c_​ for Fe/CoO remains consistent between the two samples, their absolute *H*_c_​ values differ, likely due to substrate-to-substrate variations, such as differences in defect density or the distribution of polar domains. These variations can influence domain wall motion and pinning, ultimately affecting the coercivity. Importantly, for both samples, the *H*_c_ curve exhibits clear hysteresis-like behavior, as evidenced by the two distinct coercivity ​states at zero electric field. This demonstrates a non-volatile effect, where the application of an appropriate positive or negative electric field exceeding a certain threshold, induces a remnant state with high or low coercivity, respectively. As seen in Fig. 3a, orange and red curves, the difference between the low and high coercivity states weakens with increasing temperature, and it disappears entirely above the T_B_ of CoO (see Fig. 3b–d for the comparison of hysteresis loops measured after applying positive and negative electric field of 6 kV/cm at each temperature). This reveals that the CoO in the AFM state is essential for achieving non-volatile electric-field-induced modifications in the magnetic properties of the investigated system. Additionally, for part of the sample “B” with the Fe layer grown on Cr/PMN-PT we did not observe any changes in coercive field or in magnetic anisotropy after application of an electric field (see Supplementary Material Fig. S5). This suggests that the piezoelectric-driven modulation of magnetic properties in Fe/CoO is mediated by changes in the magnetic state of the CoO film.

It should be noted that the difference between the two coercivity states observed at 80 K in Fig. 3a is reduced compared to the difference between the virgin state and the post-electric state at the same temperature, as shown in Fig. 2b. This may result from the tendency of PMN-PT substrates to undergo non-reversible deformation of the crystal lattice after the first application of an electric field along the [001] direction, which triggers the reorientation of the randomly oriented polar domains into a multidomain 4R state^8,34^. Consequently, electric-field-induced changes in magnetic properties related to initial poling of the substrate may be irreproducible.

**Fig. 4:**
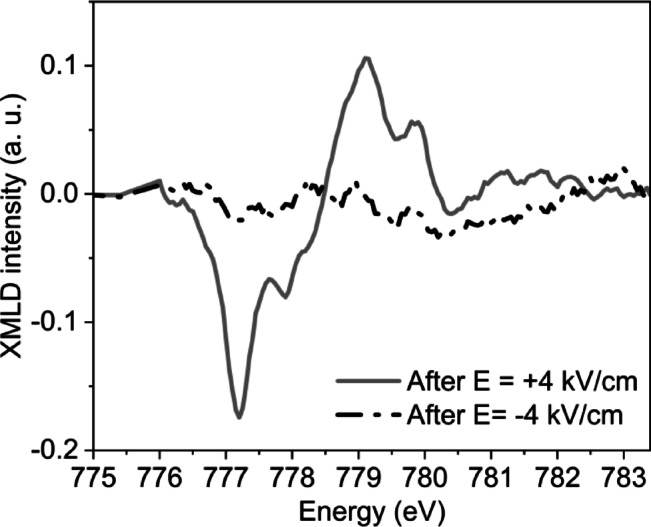
XMLD signal of CoO obtained at 80 K after the application of a negative (black dashed line) and positive (grey line) electric field across the PMN-PT substrate.

To directly demonstrate the effect of an electric field on the reorientation of AFM spins, we performed XMLD measurements on a sample without a ferromagnetic capping layer. Measurements were performed at 80 K after applying electric fields of E = − 4 kV/cm and E = + 4 kV/cm across the PMN-PT substrate at room temperature. Figure 4 presents the resulting XMLD signal, calculated as the difference between XAS spectra recorded at normal and grazing incidence (see Supplementary Materials Fig. S6 for details). No XMLD signal was detected following the application of a + 4 kV/cm field (Fig. 4, black dashed line). However, a pronounced XMLD response appeared when the polarity was reversed (Fig. 4, grey line). Although the polycrystalline nature of the CoO layer prevents precise determination of the AFM spin orientation, a change of the XMLD signal with electric field polarity provides direct evidence that the AFM spin structure is responsive to electric-field-induced strain. These XMLD results confirm that rotatable AFM spins in CoO are sensitive to electric fields applied via the PMN-PT substrate.

The observed strain-induced reorientation of AFM spins can be attributed to several mechanisms, with a primary factor being the modification of magnetic anisotropy (MA). This strain-induced alteration of MA may arise from changes in orbital occupation, crystal field splitting, or spin-orbit interactions^35–37^. In FM/AFM bilayers, strain can also influence the relative proportion of rotatable and pinned AFM spins^38^, thereby affecting the exchange bias and coercivity of the system. In our study, we observed a dependence of coercivity on the electric field, along with a minor change in exchange bias after the initial application. However, no significant or systematic electric-field sensitivity of the exchange bias was detected. Thus, we do not attribute the observed effects to a change in the relative population of pinned versus rotatable AFM spins. Instead, the strain-induced variation in the XMLD signal is more likely a consequence of electric-field-induced modification of magnetic anisotropy in CoO. Since magnetic anisotropy is the key factor stabilizing the AFM domain pattern, its strain-induced modification can be responsible for transformations of the AFM domain structure in CoO.

**Fig. 5:**
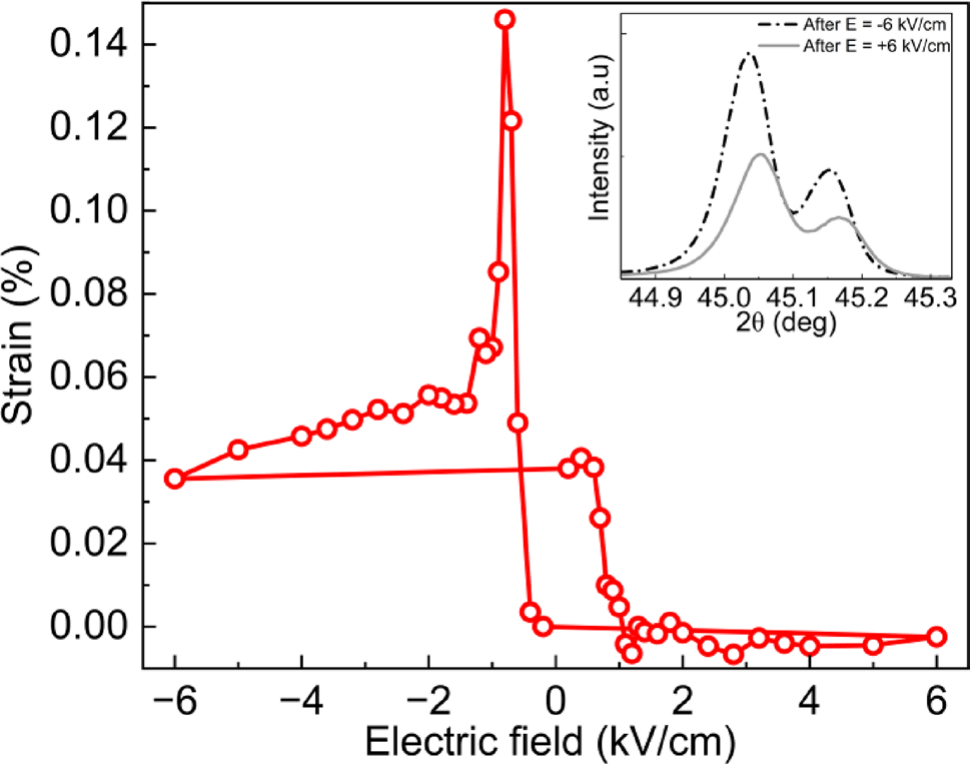
Remanent out-of-plane strain as a function of the electric field applied along the [001] direction to the single-crystal PMN-PT(001) substrate. Each data point is derived from the room temperature XRD pattern, taken at zero electric field, after applying the specified electric field. The inset shows XRD scans of the (002) reflection with CuKα1-CuKα2 profile of the PMN-PT substrate upon removing electric fields of + 6 kV/cm (gray curve) and − 6 kV/cm (black curve).

To demonstrate that the modulation of the magnetic properties of the Fe/CoO bilayer is linked to the piezoelectric response of the substrate, we performed XRD measurements. The electric field-induced changes in out-of-plane lattice parameter of the PMN-PT substrate are reflected in the shift of the (002) reflection, as described by the Bragg’s law (see inset of Fig. 5). To confirm the presence of non-volatile strain, we measured XRD scans of the (002) PMN-PT reflection at zero electric field following the application of an electric field. The remanent out-of-plane strain is calculated with respect to the positively poled sample. As seen in Fig. 5, the remanent out-of-plane strain exhibits distinct high and low states upon the application of sufficiently high negative and positive electric fields, respectively. When decreasing the electric field from E = 0 kV/cm to E = − 6 kV/cm, a pronounced peak appears at E = − 0.8 kV/cm, corresponding to 0.14% elongation of unit cell along the PMN-PT [001] direction. Further, the strain gradually decreases until the maximum negative electric field is reached. Upon reversing the polarity and increasing the electric field from E = 0 kV/cm to E = + 6 kV/cm, the strain initially remains constant but then sharply decreases after reaching E = + 0.6 kV/cm, as the lattice returns to its initial state. These results confirm that remanent strain can be induced in the system through the application of an electric field. Under the assumption of approximate volume conservation via the Poisson effect, changes in the out-of-plane lattice constant are expected to be accompanied by variations in the in-plane lattice parameters^17,39^ which, in turn, enable non-volatile modifications of the magnetic properties in Fe/CoO bilayers. Remanent strain in the (001)-oriented PMN-PT substrates has been previously observed and attributed to the 109° ferroelastic domain switching^7,40^.

The asymmetry in the strain response to the applied field observed in Fig. 5 may be related to the presence of defect dipoles within the ferroelectric material^41^. Defect dipoles locally interact with ferroelectric domains, making their reorientation more difficult in one direction than in the other and generating an internal bias field that influences domain switching behavior^42^. Since the polarization of the ferroelectrics is directly related to its piezoelectric response, defect dipoles can affect strain behavior. Besides defects, the mobility of polar domains in PMN-PT substrates might be changed upon cooling due to the so-called freezing effect in relaxor ferroelectric. As shown in Ref.^43^, even cooling the system by 50 K below room temperature makes the polar domains in PMN-PT more difficult to rotate, requiring a higher electric field for switching compared to room temperature. We assume that the freezing effect plays a role in our system, explaining why a much higher electric field is required to switch the coercivity state in the Fe/CoO bilayer at 80 K (Fig. 3, blue triangles), than it would be expected based on the remanent strain vs. electric field dependence measured at room temperature (Fig. 5). This interpretation could be further supported by the fact that at 230 K, a lower electric field is sufficient to induce changes in *H*_c_, compared to the measurements taken at 80 K (Fig. 3a).

## Conclusions

To summarize, we investigated the magnetic properties of polycrystalline Fe/CoO bilayers grown on piezoelectric PMN-PT(001). We observed a significant reduction in the coercive field after the initial application of an electric field across the piezoelectric substrate, compared to the virgin state. Subsequent voltage applications induced reversible, non-volatile changes in the coercive field of Fe/CoO/Cr below the Néel temperature of CoO. In contrast, no changes in magnetic properties were observed for Fe/Cr/PMN-PT. XMLD measurements confirmed that the electric field-induced non-volatile changes in the magnetic properties of the bilayer are associated with modifications in the magnetic anisotropy of the AFM film. Replacing CoO with an AFM material with a higher Néel temperature, such as NiO, could enable non-volatile, room-temperature control over the AFM state, offering potential for low-power, AFM-based spintronic writing applications.

## Supplementary Information

Below is the link to the electronic supplementary material.

Supplementary Material 1

## Data Availability

The data that support the findings of this study are available from the corresponding author upon reasonable request.
