# Supplementary material for: Control of non-volatile magnetic properties of Fe/CoO grown on a piezoelectric substrate
Source: Sci Rep. 2025 Dec 10;16:1276. doi: 10.1038/s41598-025-31017-x (PMC12789066; doi:10.1038/s41598-025-31017-x)
Supplement: Supplementary file 1 — Supplementary Material 1 [file 41598_2025_31017_MOESM1_ESM.pdf]

Supplementary Material

Control of non-volatile magnetic properties of Fe/CoO grown on a piezoelectric substrate

W. Janus[1,a,*] , M. Szpytma[1,b], E. Oleś[1], A. Kwiatkowski[1], P. Dróżdż[1], J. Kanak[2], M. Zając[3], M. Ślęzak[1], T. Ślęzak[1], A. Kozioł-Rachwał[1]

[1] *AGH University of Krakow, Faculty of Physics and Applied Computer Science, Krakow, Poland*

[2] *AGH University of Krakow, Institute of Electronics, Krakow, Poland*

[3] *National Synchrotron Radiation Centre SOLARIS, Jagiellonian University, Krakow, Poland*

[a] *current address: Institut de Ciència de Materials de Barcelona (ICMAB-CSIC), Campus de la Universitat Autònoma de Barcelona, 08193 Bellaterra, Spain*

[b] *current address: National Synchrotron Radiation Centre SOLARIS, Jagiellonian University, Krakow, Poland*

* *contact author: wjanus@icmab.es*

The structural properties of the sample were characterized using an X-ray diffractometer (XRD, X'Pert MPD) equipped with Cu Kα radiation. Figure S1 shows X-ray diffraction pattern of the sample for which (001) peaks of PMN-PT were highlighted. The absence of additional peaks suggests that the evaporated layers may not exhibit an epitaxial nature.

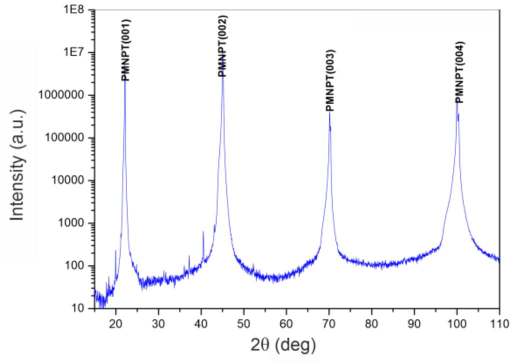

Figure S1. XRD scan of the Fe/CoO/Cr/PMN-PT(001) system.

The magnetic properties of Fe/CoO/Cr/PMN-PT(001) were characterized using the longitudinal magneto-optic Kerr effect (LMOKE). Azimuthal angular-dependent MOKE measurements (Fig. S2) revealed negligible anisotropy in the system.

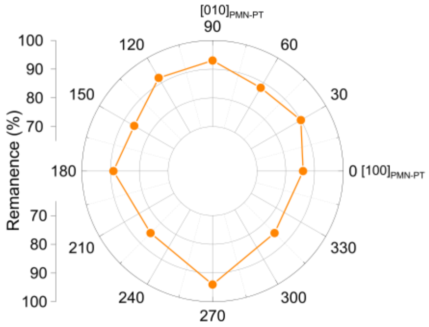

Figure S2. In-plane angular dependence of the magnetic remanence determined from MOKE hysteresis loops measured for Fe/CoO/Cr/PMN-PT(001).

Figure S3 shows hysteresis loops measured after both field-cooling (FC) and zero-field-cooling (ZFC) procedures. The blue and red curves correspond to the first and second hysteresis loops recorded after cooling the sample from 330 K to 80 K under a negative magnetic field of 1000 Oe. The horizontal shift of the loops is opposite to the field-cooling direction, consistent with a negative exchange bias. Importantly, both loops exhibit identical coercivities and exchange bias fields demonstrating the absence of a training effect in the system. Furthermore, the black curve, corresponding to the loop measured at 80 K after ZFC, shows the same H_c and H_{EB} values as those obtained after FC. This reveals the existence of a zero-field-cooling-induced exchange bias, in which the direction of H_{EB} can be determined by the remanent state of the ferromagnetic layer.

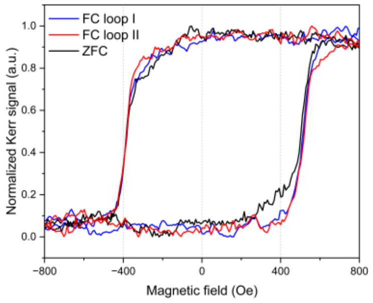

Figure S3. Hysteresis loops measured after cooling the sample from 330 K to 80 K under a magnetic field of H = –1000 Oe (field cooling, FC). The first loop is shown in blue and the second in red. The black curve represents the loop measured after zero-field cooling (ZFC).

Figure S4 shows the hysteresis loops measured for the virgin state and after the first application of an electric field at 80 K (Fig. S4(a)) and at 300 K (Fig. S4(b)). The loops measured at low temperature show

a significant difference in coercive field (H_c), indicating a strong electric-field-induced modification. In contrast, the loops measured at 300 K are nearly identical and do not exhibit the exchange bias effect.

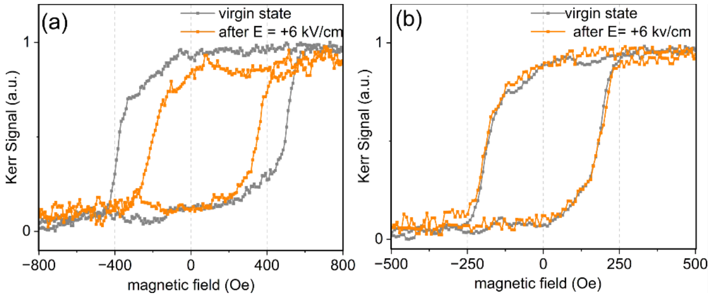

Figure S4. (a) MOKE hysteresis loops measured for the virgin state (grey curves) and after the first electric field application (orange curves) at (a) 80 K and (b) 300 K.

MOKE measurements performed on a 5 nm Fe layer grown directly on a Cr-buffered PMN-PT(001) substrate reveal no piezoelectric response in the ferromagnetic layer. No differences were observed between the LMOKE hysteresis loops obtained for Fe/Cr/PMN-PT(001) in the virgin state and after the application of either positive or negative electric field (Fig. S5(a)). Furthermore, no dependence of the H_c on the applied electric field was detected for Fe/Cr (Fig. S5(b), light blue), in contrast to Fe/CoO/Cr system, where a strong electric field dependence of H_c was observed at low temperature (Fig.S5(b), dark blue).

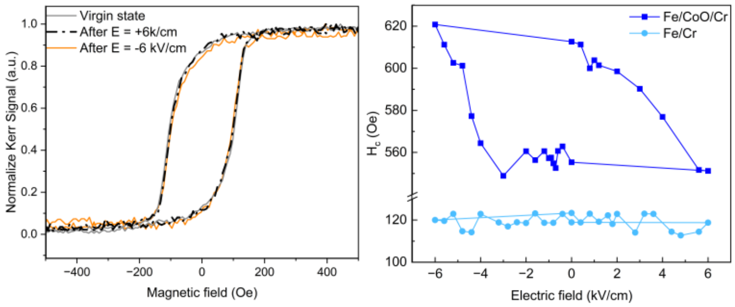

Figure S5. (a) LMOKE loops recorded at room temperature for Fe/Cr/PMN-PT(001) system with magnetic field applied along hard axis. (b) Coercive field (H_c) as a function of the applied electric field at 80 K for Fe/CoO/Cr/PMN-PT (dark blue squares) and Fe/Cr/PMN-PT (light blue circles).

The X-ray absorption spectra (XAS) were collected at normal (φ = 0°) and graizing (φ = 60°) incidence geometries. The φ defines the angle between the sample surface normal and the propagation direction of the X-rays (Fig. S6(a)). The XAS were collected at 80 K after applying electric fields of E = -4 kV/cm (Fig. S6(b)) and E = +4 kV/cm (Fig. S6(c)) across the PMN-PT substrate at room temperature. The

corresponding the corresponding X-ray magnetic linear dichroism (XMLD) signal, calculated as the difference between XAS spectra recorded at normal and grazing is shown in Fig. S6(c).

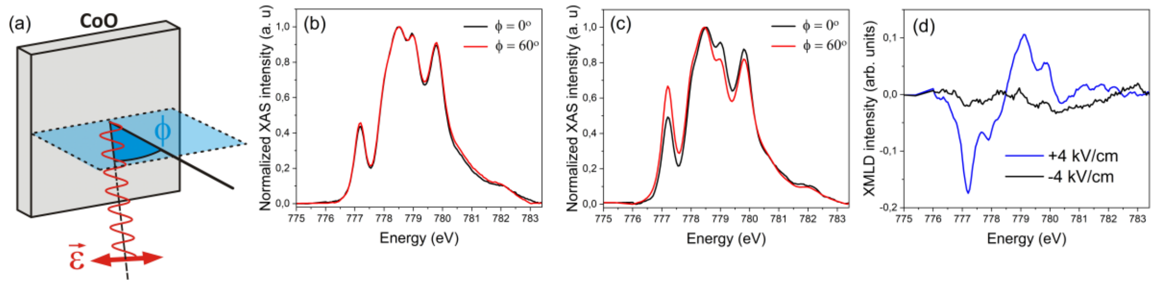

Figure S6. (a) Schematic of the geometry used for XAS measurements. (b) and (c) XAS spectra acquired after applying a negative (b) and positive (c) electric field across the PMN-PT substrate, recorded under normal (black lines) and grazing (red lines) incidence of X-rays. (d) XMLD signal obtained at 80 K after the application of a negative (black line) and positive (blue line) electric field across the PMN-PT substrate.
